# Lessons from a Mouse Model Characterizing Features of Vascular Cognitive Impairment with White Matter Changes

**DOI:** 10.4061/2011/978761

**Published:** 2011-11-09

**Authors:** Masafumi Ihara, Hidekazu Tomimoto

**Affiliations:** ^1^Department of Neurology, Kyoto University Graduate School of Medicine, 54 Kawahara-cho, Shogoin, Sakyo, Kyoto 606-8507, Japan; ^2^Department of Neurology, Mie University Graduate School of Medicine, 2-174 Edobashi, Tsu, Mie 514-8507, Japan

## Abstract

With the demographic shift in age in advanced countries inexorably set to progress in the 21st century, dementia will become one of the most important health problems worldwide. Vascular cognitive impairment is the second most common type of dementia after Alzheimer's disease and is frequently responsible for the cognitive decline of the elderly. It is characterized by cerebrovascular white matter changes; thus, in order to investigate the underlying mechanisms involved in white matter changes, a mouse model of chronic cerebral hypoperfusion has been developed, which involves the narrowing of the bilateral common carotid arteries with newly designed microcoils. The purpose of this paper is to provide a comprehensive summary of the achievements made with the model that shows good reproducibility of the white matter changes characterized by blood-brain barrier disruption, glial activation, oxidative stress, and oligodendrocyte loss following chronic cerebral hypoperfusion. Detailed characterization of this model may help to decipher the substrates associated with impaired memory and move toward a more integrated therapy of vascular cognitive impairment.

## 1. Introduction

Subcortical ischemic vascular dementia (SIVD) is characterized by white matter (WM) changes and lacunar infarctions, which occur as a result of a reduction in cerebral blood flow (CBF) over an extended period of time, causing small vessel changes [1–3]. Cerebrovascular WM lesions, neurodegenerative manifestations characterized by hyperintense signals on magnetic resonance images, are frequently associated with aging and are responsible for the cognitive decline in the elderly population [1–7]. Chronic cerebral hypoperfusion is likely to cause such WM lesions as CBF is decreased in these patients [2, 8]; indeed, similar WM lesions can be induced in rats, gerbils, and mice after chronic cerebral hypoperfusion, with experimental conditions mimicking chronic cerebral ischemia in humans [9–11]. These model animals can be generated by bilateral common carotid artery (CCA) occlusion in rats (2-vessel occlusion (2VO)) [9, 12, 13] or in mice [14], bilateral CCA stenosis in mice (BCAS) [10] or in gerbils [11], and unilateral CCA occlusion in mice [15]. Nonhuman primates appear to represent the best model for the study of WM lesions, because they have well-developed WM and vascular architectures which closely resemble those in human brains [16]. Nevertheless, most experiments studying chronic cerebral hypoperfusion have been performed in rodents because of the ease of handling and higher acceptability from an ethical viewpoint. 

The rat model of chronic cerebral hypoperfusion is accompanied by cognitive impairment and cholinergic deficits [9, 13, 17] and is most widely used [12, 18]. These animals develop WM rarefaction [9, 19], which appears very similar to that found in human cerebrovascular WM lesions. However, this model has some drawbacks. For example, the visual pathway is injured by the occlusion of the ophthalmic arteries and thus may compromise behavioral assessment. Furthermore, genetic studies may be hampered because of limited accessibility to molecular technologies when using knockout or transgenic animals. To circumvent such limitations, we have established a mouse model of chronic cerebral hypoperfusion, which is subjected to various degrees of CBF reduction by the narrowing of the bilateral CCAs with newly designed microcoils. The model demonstrates good reproducibility in terms of WM lesion appearance and glial activation. The cerebral WM is selectively damaged, while gray matter (including hippocampal) integrity remains intact, if the degree of stenosis is appropriately controlled by internal diameter regulation of the microcoils [10].

The aims of the current paper are to provide a comprehensive survey of the experimental evidence that has accumulated since establishment of this mouse BCAS model, in order to extrapolate the results into human neurological conditions, and to consider the particular strengths and pitfalls of the method.

## 2. The Procedures for BCAS

Ten-week-old male C57BL/6J mice (24–29 g) are conventionally used to induce chronic cerebral hypoperfusion by BCAS [10, 20, 21]. This model should be applied exclusively to C57BL/6J strain, because the CBF in the other strains may have a greater variability after BCAS. In this paper, unless stated otherwise, the “BCAS mouse” indicates a male C57BL/6J mouse that is subjected to BCAS for 30 days from 10 weeks of age using microcoils of 0.18 mm in diameter. 

**Figure 1:**
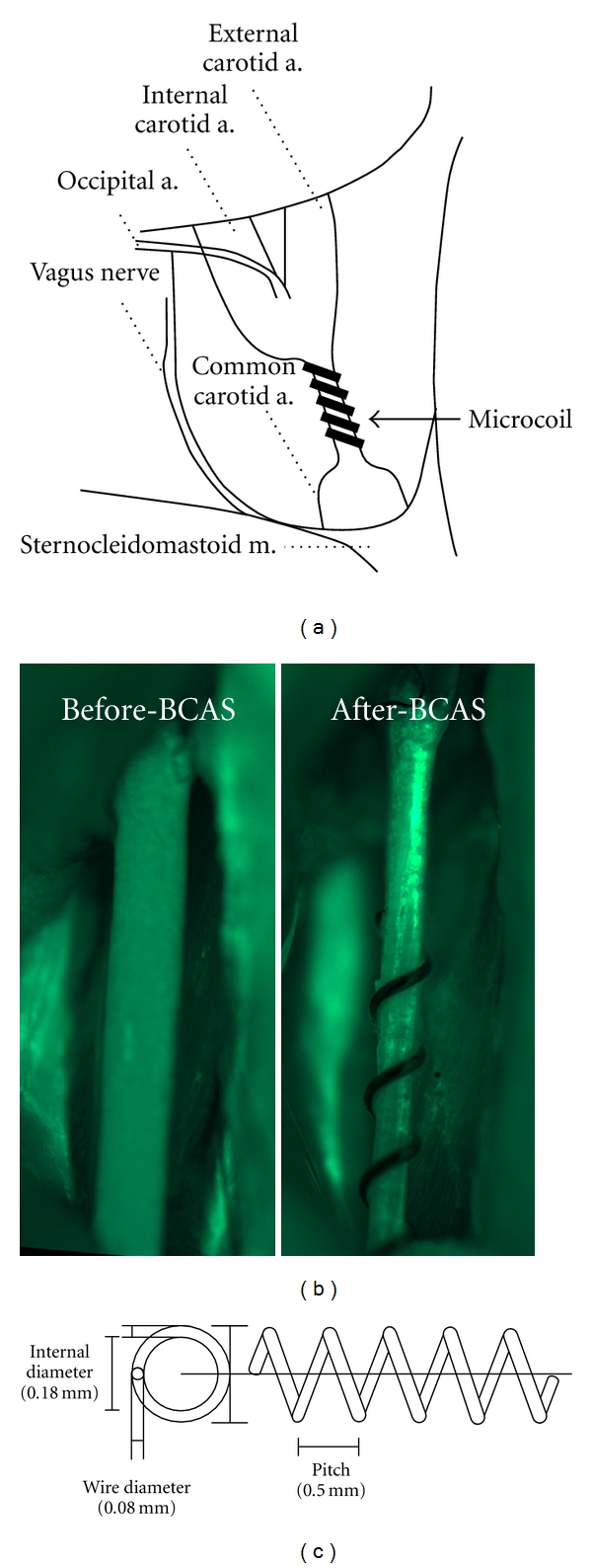
The procedure for BCAS and the microcoil. The microcoil is twined by rotating it around the CCA just proximal to the carotid bifurcation of a C57BL/6J mouse (a). Representative photographs of a FITC-perfused common carotid artery before (left) and after (right) placement of a microcoil (b). The microcoil is made from piano wire (wire diameter of 0.08 mm) with an inner diameter of 0.18 mm, pitch 0.50 mm, and total length 2.5 mm (c).

Mice are anesthetized with 2% halothane or 25–50 mg/Kg sodium pentobarbital and, through a midline cervical incision, both CCAs are exposed and freed from their sheaths. Two 4–0 silk sutures are placed around the distal and proximal parts of the right CCA. The artery is then gently lifted by the sutures and placed between the loops of the microcoil just proximal to the carotid bifurcation (Figures 1(a) and 1(b)). The microcoil is twined by rotating it around the CCA, and another microcoil of the same size is twined around the left CCA after 30 minutes (Figures 1(b) and 1(c)). Four types of microcoils made from piano wire with varying inner diameters from 0.16 mm to 0.22 mm (Figure 1(c)) have been designed in collaboration with Sawane Spring Co., Ltd. (Hamamatsu, Japan). Microcoils with the same diameter are conventionally placed on the bilateral CCA, though a modified model has also been devised where the 0.16 mm microcoil is placed on the left CCA and the 0.18 mm microcoil on the right CCA [22]. The rectal temperature should be maintained between 36.5°C and 37.5°C, and the cessation of CBF for >1 minute should be avoided. All procedures for BCAS are usually accomplished within 15 minutes except an interval for 30 minutes. 

### 2.1. Blood Pressure

The blood pressure of the surviving mice does not change significantly at any postoperative intervals until 30 days, compared with the sham-operated controls [10]. 

### 2.2. Mortality Rates

The mortality rates are reported to range from 10% to 20%: 13% in mice with microcoils of 0.22 mm in diameter, 17% in those of 0.20 mm, and 15–19% in those of 0.18 mm [10, 23]. In contrast, 75% (15/20) of mice with microcoils of 0.16 mm placed died within 14 days after the surgery, most of whom were found to have cerebral infarctions [10]. In another study of a modified model with the 0.16 mm microcoil on the left CCA and the 0.18 mm microcoil on the right CCA, the mortality rate is reported to be 18.8% [22].

### 2.3. Body Weight

Body weight has been shown to decrease after the surgery, but recover to baseline by day 7, in mice with 0.22, 0.20, and 0.18 mm diameter microcoils. Although the mice with the 0.22, 0.20, and 0.18 mm diameter microcoils placement tended to have a lower body weight than those with sham operation, no significant difference is noted at any postoperative interval. In contrast, the mice with the 0.16 mm diameter microcoil placement showed significantly lower body weight at all postoperative intervals, compared with the sham-operated mice [10].

### 2.4. Neurological Deficits

After placement of the 0.22, 0.20, and 0.18 mm diameter microcoils, the animals regained consciousness within a few hours and occasionally showed transient ptosis but no apparent motor weakness. In contrast, some of the mice with 0.16 mm diameter microcoils placed (~35%) did not regain consciousness, showing rolling or circling movements lasting 2 to 6 hours after awakening, and severe akinesia with a squatting posture [10].

### 2.5. Anesthetics

Although anesthetics such as sodium pentobarbital and halothane are known to provide varying degrees of neuroprotection against ischemic injury [24], the selection of anesthesia did not appreciably affect the mortality rates, temporal profile of CBF, and ischemic WM changes after BCAS.

## 3. The Spatial and Temporal Profiles of Cerebral Blood Flow and Metabolism after BCAS

Although 2VO rats develop specific WM changes without any apparent gray matter changes [9, 12], 2VO in mice will inevitably lead to a severe drop in CBF due to underdeveloped posterior communicating arteries [25]. Therefore, in mice, carotid stenosis, but not occlusion, is required to achieve cerebral hypoperfusion and resultant WM changes. CBF and cerebral metabolism change dynamically following the BCAS operation, with CBF dropping immediately after carotid stenosis and recovering over a period of months via compensatory and adaptive mechanism (i.e., collateral anastomosis).

**Figure 2:**
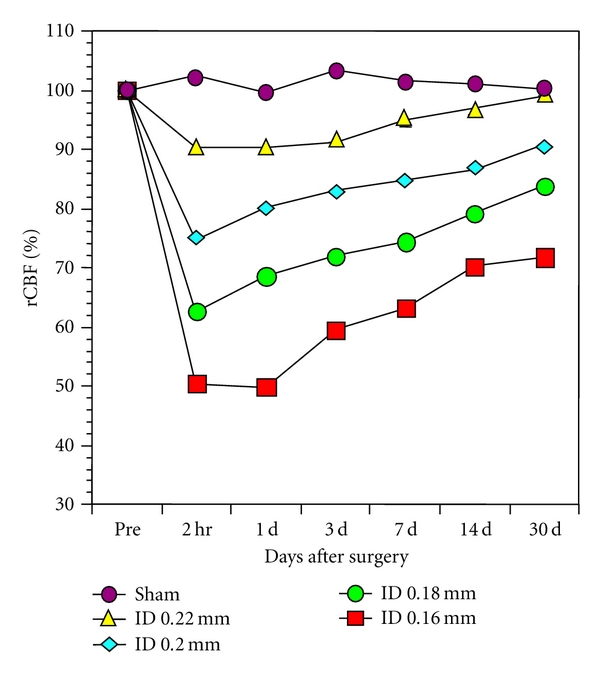
Cerebral blood flow after BCAS. This figure shows cerebral blood flow evaluated with laser Doppler flowmetry in mice at 2.5 months of age after the surgery using microcoils with diameter of 0.16 mm, 0.18 mm, 0.20 mm, and 0.22 mm. The data represent mean values expressed as a percentage of the preoperative value.

Figure 2 shows mean CBF values evaluated with laser Doppler flowmetry in surviving mice of 2.5 months of age after application of four types of piano wire with varying inner diameters, varying from 0.16 mm to 0.22 mm, to the bilateral CCAs [10]. The CBF values decreased significantly from the preoperative baseline after the surgery with the 0.20, 0.18, and 0.16 mm diameter microcoils. At 2 hours, there was a significant reduction in CBF values in mice with the 0.20 mm microcoils to 77.3 ± 13.4% (mean ± SD), 67.3 ± 18.5% in those with 0.18 mm, and 51.4 ± 11.5% in those with 0.16 mm. On day 1, the CBF values started to recover but remained significantly lower until 14 days after placement of the microcoils, compared with the control group. At 30 days, CBF values were still decreased in mice with 0.16 mm microcoils placed. Intergroup differences in CBF values were detected between mice with 0.16 mm microcoils but there were no differences among the mice with 0.22, 0.20, and 0.18 mm microcoils placed. 

Older 4-month-old mice showed a similar profile of CBF changes after the BCAS operation with the 0.18 mm coils; the CBF values temporarily decreased to 62.9 ± 18.5% (mean ± SE) at 2 hours after BCAS, compared to the sham group but gradually recovered to 81.7% ± 4.0% at 1 month, 83.2% ± 1.8% at 2 months, and 85.0% ± 8.7% at 3 months [26]. Interestingly, this temporal profile of CBF is similar to that of the first 5-minute ^18^F-fluorodeoxyglucose (FDG) uptake in the cerebral cortex, suggesting that the early ^18^F-FDG uptake scan can serve as an estimate of CBF. The early ^18^F-FDG uptake scan in the striatum showed a similar temporal profile to that of the cerebral cortex, suggesting that the CBF values in the cerebral cortex and the striatum change in parallel after BCAS. By contrast, the CBF in the hippocampus did not decrease at 2 hours or 2 months after BCAS but finally decreased at 6 months. The late ^18^F-FDG scans show that the glucose uptake in the hippocampus did not decrease by 2 months after BCAS but decreased by 20% at 6 months after BCAS. The lack of reductions in hippocampal CBF and metabolism in the early phase after BCAS is probably due to the hippocampus being supplied by both anterior and posterior circulations [27]. However, mild cerebral ischemia of an insufficient magnitude for 6 months has been shown to induce subacute pathologies, which may lead to subsequent changes in the gray matter, including the cerebral cortex and hippocampus [26]. 

## 4. Blood-Brain Barrier Disruption after BCAS

A previous study on the rat 2VO system and human material implicated a dysfunction of the blood-brain barrier (BBB), perivascular edema, and microglial activation as the mechanisms underlying the WM lesions [9, 28, 29]. During this process, microglia may play a pivotal role; both microglia activation and WM lesions have been shown to occur concurrently, and both are suppressed by the administration of immunosuppressants, such as cyclosporin A or FK 506 [30, 31]. Proteases derived from the microglia may contribute to the reduction in the basement membrane components and BBB breakdown [32, 33]. The resulting perivascular edema may further exacerbate the degradation of the WM myelin through the actions of extravasated serum factors [28]. The BBB breakdown also leads to leukocyte diapedesis [34], and the infiltrating leukocytes may cause inflammatory demyelination. Matrix metalloproteinase-2 (MMP-2), through its activity as a type IV collagenase, is activated and degrades components of the basement membrane. In addition, MMP-2 has been shown to degrade myelin basic protein at approximately 100x more potency than MMP-9 [35]. Thus, the MMP-2 released from glial cells may be directly involved in the remodeling of WM myelin [36].

Consistent with the aforementioned notion, BBB disruption has been shown to be accompanied by an upregulation of MMP-2, but not MMP-9, suggesting the specific involvement of MMP-2 in WM lesion manifestation in the 2VO rat model [37]. In rats treated with a relatively selective MMP-2 inhibitor, AG3340, the WM lesions become significantly less severe after chronic cerebral hypoperfusion, and the density of activated astroglia and microglia significantly reduced, compared with the vehicle-treated rats [21]. Gene knockout of MMP-2 also reduced the severity of WM lesions and the density of activated astroglia and microglia in a mice BCAS system. In both rodents, disruption of BBB function, as assessed by IgM staining and the Evans blue extravasation test, was less severe when MMP-2 activity was attenuated. The most marked extravasation in Evans blue test, in the paramedian portion of the corpus callosum facing the lateral ventricle in the BCAS mouse, is consistent with a previous report on a rat model of chronic cerebral hypoperfusion [38] and further indicates a vulnerability of the BBB in this area. Rosenberg et al. showed that the activated astroglia and microglia/macrophages present around arterioles express MMP-2 and MMP-3, but not MMP-9, in the brains of patients with vascular dementia [39]. The major pathologic features of WM lesions, such as demyelination and gliosis, may result from a BBB dysfunction, which may result in the leakage of proteins and fluid through the compromised barrier of the penetrating arteries [40]. 

## 5. White Matter Injury after BCAS

**Figure 3:**
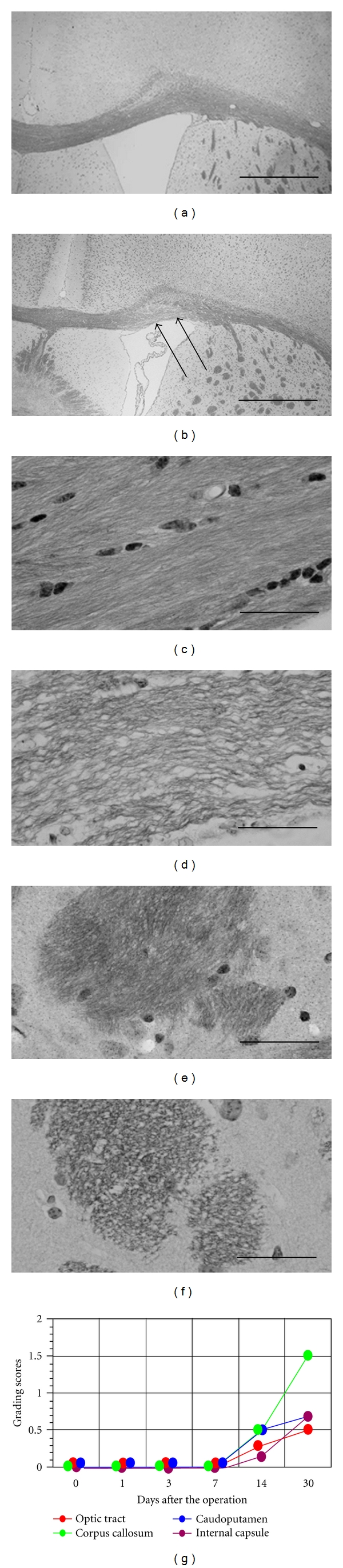
White matter changes after BCAS. Photomicrographs of Klüver-Barrera staining in the cerebral cortex (a, b), corpus callosum (c, d), and caudoputamen (e, f). The left column (a, c, e) indicates the brain from a sham-operated mouse, and the right column (b, d, f) indicates a brain after BCAS-operated mouse using microcoils with 0.18 mm diameter for 30 days. Note that the WM changes are the most intense in the medial part of the corpus callosum adjacent to the lateral ventricle (arrows). The histogram shows temporal profiles of the WM changes, the severity of which is semiquantitatively graded into four levels (g). Scale bar, 500 *μ*m (a, b), 50 *μ*m (c, d), and 25 *μ*m (e, f).

In BCAS mice with 0.18 mm microcoils placed, the temporal profile of the WM lesions was examined (Figure 3) [10, 20, 21, 41–43]. WM lesions were not detected in any region of the brain 3 and 7 days after BCAS. After 14 days, the WM lesions were evaluated as grade 0 or 1 in the medial part of the corpus callosum, caudoputamen, and the internal capsule; however, after 30 days, severe rarefaction occurred in these regions. WM lesions were most densely distributed in the medial part of the corpus callosum adjacent to the lateral ventricles; the lesions were moderately distributed in the paramedian part of the corpus callosum, fiber bundles of the caudoputamen, and the internal capsule; lesion distribution was, however, less severe in the anterior commissure and the optic tract. The staining intensity of the myelinated fibers was reduced and the integrity of the myelin compromised in the WM regions. The remaining fibers were disorganized and vacuoles frequently observed in the neuropil. There were relatively few TUNEL positive cells in the corpus callosum [20]. In contrast, the WM lesions in the optic tract did not emerge until 30 days. Atrophy was not found in the optic nerve, though there was evidence of slight rarefaction. In each region of the WM, the numerical densities of the microglia/macrophages immunolabeled for MHC class II antigen increased significantly from 7 to 30 days after BCAS whereas astroglia immunolabeled for GFAP increased in the period from 14 to 30 days and the regions with intense glial activation corresponded to those with a greater loss of WM myelin. There was a significant negative correlation between the CBF at any time point after BCAS and the grading scores of WM lesions at 30 days [10]. Thus, lower CBF appears to be present in the more severe WM injuries. This notion is further strengthened by the finding that mice with 0.16 mm microcoil on the left CCA and 0.18 mm microcoil on the right CCA exhibited more severe WM injury in the left hemisphere [22]. 

In accordance with the histological findings, *in vivo* MRI showed reductions in fractional anisotropy in the corpus callosum and internal capsule and a significant decrease in the magnetization transfer ratio in the corpus callosum, fimbria, internal capsule, and optic tract following hypoperfusion [44]. Hypoperfused mice demonstrated diffuse axonal and myelin pathology, which was essentially absent in control mice. Both fractional anisotropy and magnetization transfer ratio correlated with markers of myelin integrity/degradation and not axonal pathology. Furthermore, in a rat 2VO model, an increase in apparent diffusion coefficients on MRI was reported to be linked with MMP-2 or -9 activity and edema in WM [45]. These data therefore suggest that *in vivo* MRI is a sensitive measure of vasogenic edema and WM changes in the murine brain [44, 45].

## 6. Impairment of Learning and Memory after BCAS

In working memory tasks, tested with the 8-arm radial maze, BCAS mice made significantly more errors than the control mice following one month of hypoperfusion, although they did show normal spatial reference memory in the 8-arm radial maze test [20]. Spatial reference memory task is related to cognitive domains likely to rely on the integrity of the hippocampus, and therefore preserved reference memory is in agreement with lack of histological damage in the hippocampus [10]. In contrast, working memory impairment may be attributable to either the frontal WM lesions observed and/or hippocampal damage, which is undetectable by the conventional histological methods. In previous studies, working memory deficits have been related either to the hippocampus or frontal subcortical circuits in the rodent [13, 46] and likely primates [47, 48]. Therefore, disruption of WM tracts, especially within the prefrontal cortex, may be another mechanism behind age-related changes in working memory function [49]. A previous study has also shown that there is a selective impairment in spatial working memory, with all other measures of spatial memory remaining intact, in the BCAS mice with selective WM damage [42].

In contrast, in the longer-term BCAS model, in addition to WM changes, there were also significant hippocampal changes (atrophy and cell death) documented 8 months after BCAS (see Section 7). Consistent with these histological changes, a series of behavioral batteries demonstrate deficits in both working and reference memory. Thus, longer-term hypoperfusion more accurately replicates the advanced stages of SIVD and possibly provides evidence linking chronic hypoperfusion and aging [26]. 

## 7. Neuropathologic Changes Induced by BCAS

No infarctions or hemorrhage develops in any gray matter regions in mice with the 0.22, 0.20, and 0.18 mm microcoils after 1 month of chronic cerebral hypoperfusion [10]. There are no TUNEL positive neurons in the hippocampus [20]. In contrast, more than half of the BCAS mice with 0.16 mm microcoils placed exhibited microinfarcts in the parietal cortices, neuronal loss in the CA1 subfield of the hippocampus, and patchy necrotic lesions in the caudoputamen [10]. 

 In contrast, at 8 months after BCAS, pyknotic neurons have been frequently observed in the cerebral cortex and the hippocampus. Furthermore, significant atrophy has been noted in the hippocampus but not in the cerebral cortex or the corpus callosum. The number of fragmented or shrunken nuclei stained for single-stranded DNA increased in the CA1 and CA3 sectors of the hippocampus but not in the dentate gyrus. 

Given that the shorter-term (conventional) BCAS mice demonstrate WM damage without any apparent hippocampal damage at 1 month after BCAS, hippocampal degeneration in the longer-term BCAS mice may be secondary to the preceding WM damage. This may then subsequently contribute to the dementia syndrome, partly overlapping with Alzheimer's disease (AD) in their cognitive profiles and histological changes. In probable AD patients, a linear relation is found between WM lesions and hippocampal atrophy on MRI, especially for WM lesions in the frontal and parietooccipital regions [50]. A disconnection of the hippocampus by cerebrovascular WM lesions in the white matter tracts subserving the cortical association areas may lead to shrinkage of the hippocampus due to Wallerian degeneration as the hippocampus receives most of the input from the neocortical association cortices [51]. These findings are intriguing given the widely accepted fact that vascular dementia and AD both increase in prevalence with age, frequently occur concomitantly, and overlap considerably in their symptomatology, pathophysiology, and comorbidity [52]. WM damage may thus be one of the pathological substrates that mediates such a linkage between neurodegenerative and cerebrovascular disorders.

## 8. BCAS-Mediated Acceleration of Neurodegeneration: Linkage between Hypoperfusion and Neurodegeneration

WM attenuation has also been frequently observed in neurodegenerative disorders, such as AD and dementia with Lewy bodies [53]. MR imaging has revealed that such changes manifest as WM lesions, which increase with older age [54]; this is particularly apparent in AD and dementia with Lewy bodies, compared to ageing controls, though to a lesser extent than in vascular dementia. WM lesions in AD progress relatively slowly if a multicomponent intervention is given to reduce vascular risk factors [55], suggesting that ischemic changes underlie the WM lesions in AD. However, different mechanisms have been, at least to a certain extent, associated with myelin degeneration as it has been shown that myelin loss evolves in parallel with shrunken oligodendrocytes in vascular dementia but with their increased density in AD [56]. Further investigation is thus warranted to clarify the wider question of whether vascular brain injury has additive effects on AD pathogenesis [57–59]. To tackle this question, AD model mice have been subjected to chronic cerebral hypoperfusion by BCAS.

Biochemical analyses have indicated that BCAS increases the level of conformationally changed A*β* in soluble extracellular-enriched brain fractions in a relatively low-(J9) [60] and high-expressor line (J20) of the APP_Sw/Ind_ mouse [61]. The latter study also demonstrated that BCAS significantly reduced the density of cored plaques and neurons of the hippocampus [61]. Notably, chronic cerebral hypoperfusion and APP_Sw/Ind_ overexpression interdependently disrupted reference memory [61]. Therefore, soluble, but not insoluble, A*β* species may play a direct role in neurotoxicity and resultant behavioral abnormalities in the hypoperfused APP_Sw/Ind_ mice. Since the vascular-type lesions reproduced in the BCAS model are oligemic (e.g., noninfarctional) chronic hypoperfusion may accelerate AD neuropathology in a latent manner over an extended period of time via enhanced neuronal loss and altered A*β* metabolism. Given that oligodendrocytes are highly susceptible to A*β* toxicity [62], the results may further provide evidence linking chronic hypoperfusion with neurodegeneration.

## 9. Treatment: Future Directions on Intervention 

### 9.1. MMP Inhibitor

The MMP inhibitor AG3340 has been shown to possess protective effects against WM lesions after chronic cerebral hypoperfusion in rats [21]. AG3340 administration has been shown to decrease IgM-immunoreactive glial cell density in the vicinity of the microvessels in the corpus callosum, suggesting it helps restore BBB integrity [21, 37]. Furthermore, genetic deletion of MMP-2 has been shown to attenuate the WM lesions after BCAS in mice. These data suggest the potential value of MMP inhibitors in preventing SIVD resulting from BBB dysfunction and chronic cerebral ischemia in humans [39]. An elucidation of the exact roles of MMP-2 in BBB disruption may also provide information useful in developing strategies for controlling WM damage. 

### 9.2. Adenosine A_****2A****_ Ligand

As an endogenous neuromodulator in the brain, the extracellular levels of adenosine markedly increase under hypoxic/ischemic conditions. Adenosine exerts its physiological actions through activation of four G-protein-coupled membrane receptors, the A_1_, A_2A_, A_2B_, and A_3_ receptors [63]. The A_2A_ receptor has drawn attention for its ability to modify a variety of brain insults; for instance, mice deficient in the A_2A_ receptor have been shown to possess substantially smaller infarct volumes and better neurological behavioral deficit scores after transient focal ischemia [64]. A_2A_ receptor antagonists have also been shown to attenuate ischemic brain injury [65], suggesting a neuroprotective role of A_2A_ in acute ischemic injury. However, adenosine's action is likely to be diverse in the setting of brain injury as brain damage aggravates after hypoxic ischemia in immature A_2A_ knockout mice [66]. A recent study has further indicated that, following the BCAS operation, A_2A_ receptor knockout mice display more extensive demyelination-related damage together with proliferation of astrocytes and microglia in the WM, compared with wild-type mice [23]. Working memory, evaluated by means of an 8-arm radial maze test, is also more seriously impaired in A_2A_ receptor knockout mice relative to wild-type mice. Such effects have been associated with increased expression of proinflammatory cytokines, including tumor necrosis factor-*α*, interleukin-1*β*, and interleukin-6 in the WM. Therefore, activation of the A_2A_ receptor by its ligand may ameliorate the WM damage and cognitive deficits induced by BCAS through suppression of proinflammatory cytokines. Although the A_2A_ receptor may be a potential therapeutic target for the treatment of ischemic WM damage, a potential pitfall in their use may be their apparent opposing effects on different cell types such as neurons, inflammatory cells, and glial cells.

### 9.3. Angiotensin II Type 1 Receptor Blocker

Drugs that target the rennin-angiotensin system seem to have particular potential for prevention of dementias, including AD and vascular dementia. The Perindopril Protection Against Recurrent Stroke Study (PROGRESS) has suggested a protective effect of angiotensin-converting enzyme inhibitors on cognitive function in patients with stroke [67]. Moreover, the Study on Cognition and Prognosis in the Elderly (SCOPE) trial demonstrated a positive effect of the angiotensin II type 1 receptor blocker (ARB), candesartan, in a subgroup of elderly hypertensive patients with mild cognitive impairment [68]. Notably, a prospective cohort analysis of 819 491 participants has suggested that ARBs are associated with a significant reduction in the incidence and progression of dementia, even compared with angiotensin-converting enzyme inhibitors [69].

In accordance with the above clinical findings, telmisartan, an ARB with unique “delta lock” structure that strongly binds to angiotensin II type 1 receptor [70], and possesses a high degree of lipophilicity and thus the ability to cross the blood-brain barrier [71], has been shown to exert protective effects against WM damage and cognitive impairment in the BCAS mice [41]; it is thought to achieve this by alleviating microglial/astroglial activation, endothelial oxidative stress, and oligodendrocyte loss [41]. Notably, such protective effects are observed with a nonhypotensive dose, but not with a hypotensive dose of telmisartan, suggesting that such protective effects against WM lesions are independent of blood pressure, and are at least partially mediated by anti-inflammatory and antioxidative effects that are exerted in part by the pleiotropic effects of telmisartan such as PPAR-*γ* activation [41, 71]. Thus, telmisartan may be considered as a putative treatment for SIVD, though caution should be exercised when lowering blood pressure if cerebrovascular autoregulation is damaged. In clinical practice, appropriate timing and dose of telmisartan should be considered.

### 9.4. Adrenomedullin

Adrenomedullin (AM) has a variety of effects on the vasculature that include vasodilation, regulation of permeability, inhibition of endothelial cell apoptosis and oxidative stress, regulation of smooth muscle cell proliferation, and promotion of angiogenesis [72, 73]. AM heterozygosity in mice resulted in increased infarct volume with significant accumulation of inducible nitric oxide, oxidative DNA damage, and lipid peroxidation after transient focal ischemia [74] whilst prophylactic administration of AM alleviated cerebral edema in the striatum and cerebral cortex in a rat stroke model [75]. In BCAS mice, increased levels of circulating AM have been shown to restore cerebral hemodynamics, promote arteriogenesis, as well as angiogenesis, alleviate oxidative damage in cerebral microvessels, and preserve WM integrity; importantly, this subsequently attenuates working memory deficits in an 8-arm radial maze test [43]. In addition, AM selectively upregulates brain levels of cyclic AMP, vascular endothelial growth factor, and basic fibroblast growth factor in the hypoperfused, but not the normoperfused, brain. Furthermore, proangiogenic/arteriogenic changes did not occur in sham-operated AM-overexpressing mice where the expression of AM receptor component RAMP2 is significantly suppressed, possibly through feedback inhibition. Such tissue selectivity could be an advantage for clinical application of AM in patients with SIVD; AM-induced arteriogenesis and angiogenesis could be induced only in hypoperfused tissue. 

### 9.5. Bone Marrow Transplantation

Therapeutic use of bone-marrow-derived cells has been shown to ameliorate functional deficits after stroke and is accompanied by augmentation of angiogenic and regenerative responses [76]. Although early functional improvement has been noted within days of treatment, its precise mechanism remains to be elucidated. A recent study has demonstrated that administration of bone marrow mononuclear cells (BMMNCs) induces immediate endothelial nitric oxide synthase-dependent vasodilation in ischemic femoral arteries [77]. 

In BCAS mice, BMMNC treatment has been shown to provide strong protection against WM damage, dependent primarily on CBF recovery beginning from the early phase, and the subsequent endogenous restorative response, including angiogenesis, in a later phase [78]. Both of these responses involve nitric oxide synthase activation. Despite marked protection against WM damage, no direct structural incorporation of donor BMMNCs to oligodendrogenesis was found, although a fraction of donor cells were found to wrap around the microvessels with features suggestive of pericytes [78, 79]. While a direct antiapoptotic effect on oligodendrocytes may be involved in the WM protection [80], it is plausible that CBF recovery after BMMNC treatment is sufficient to maintain WM integrity. Additional investigation is therefore required to assess whether other mechanisms such as direct structural incorporation or direct antiapoptotic effect of BMMNCs play a role in the WM protection. The above findings suggest clinical applicability of BMMNC treatment for SIVD management. 

## 10. A New Concept of “Oligovascular Niche”

**Figure 4:**
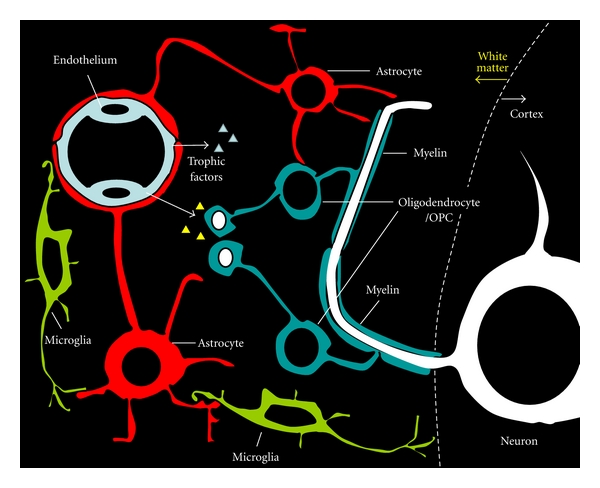
A schematic illustration of oligovascular niche. In the “oligovascular niche,” crosstalk between endothelial cells and oligodendrocytes mediated by an exchange of soluble signals (e.g., trophic factors or chemical messengers) might play an important role in sustaining oligodendrocyte homeostasis and WM integrity. Since oxidative stress and inflammation caused by cerebral hypoperfusion would be detrimental for this niche, maintenance of white matter integrity or oligovascular protection could be achieved by proangiogenic, antioxidative, and anti-inflammatory interventions. OPC: oligodendrocyte precursor cell.

Recently, the concept of “oligovascular niche” has been proposed, where crosstalk between the endothelial cells and oligodendrocytes mediated by an exchange of soluble signals (such as brain-derived neurotrophic factor and fibroblast growth factor) might play an important role in sustaining oligodendrocyte homeostasis and WM integrity (Figure 4) [81, 82]. In the oligovascular unit, endothelial cells release trophic factors that promote oligodendrocyte precursor cell proliferation. Noncytotoxic levels of oxidative stress or blockade of Src and Akt signaling prevents endothelial trophic factors from supporting oligodendrocyte precursor cells. Therefore, to treat or prevent WM damage in SIVD, endothelial cells and oligodendrocytes should be protected from being damaged due to hypoperfusion.

 Since cerebral endothelial cells contribute to numerous signaling cascades that help regulate brain homeostasis and function [83], angio-/arteriogenesis and inhibition of oxidative damage in the cerebral endothelial cells might lead to oligovascular protection—namely, successful vascular growth and vasoprotection and preservation of WM/oligodendrocyte integrity—and prevention of cognitive decline after chronic cerebral hypoperfusion. Therefore, the application of proangiogenic, antioxidative, and anti-inflammatory factors, including the aforementioned drug- and cell-based therapy, may offer potential for the treatment of WM changes and SIVD.

## 11. Summary and Conclusions

The BCAS model characterizing features of vascular cognitive impairment with WM changes may serve as a powerful tool for investigation of the molecular pathology of WM lesions and in the design of therapeutic measures for WM changes induced by chronic cerebral hypoperfusion. Although these models do not (and no other current models can) describe all features of SIVD [84], the BCAS model may help further elucidate the mechanism by which WM pathology and dementia progress in the elderly. Such knowledge may significantly enhance strategies to tackle these disorders.
